# Population Structure of *Bartonella henselae* in Algerian Urban Stray Cats

**DOI:** 10.1371/journal.pone.0043621

**Published:** 2012-08-30

**Authors:** Naouelle Azzag, Nadia Haddad, Benoit Durand, Elisabeth Petit, Ali Ammouche, Bruno Chomel, Henri-Jean Boulouis

**Affiliations:** 1 Ecole Nationale Supérieure Vétérinaire d’Alger, El Harrach, Alger, Algérie; 2 Université Paris-Est, Ecole Nationale Vétérinaire d’Alfort, UMR BIPAR ENVA Anses UPEC USC INRA, Maisons-Alfort, France; 3 Anses, Laboratoire de Santé Animale, Unité d’Epidemiologie, Maisons-Alfort, France; 4 Ecole Nationale Supérieure d’Agronomie, Département de Technologie Alimentaire, El Harrach, Alger, Algérie; 5 Department of Population Health and Reproduction, School of Veterinary Medicine, University of California Davis, Davis, California, United States of America; Institut National de la Recherche Agronomique, France

## Abstract

Whole blood samples from 211 stray cats from Algiers, Algeria, were cultured to detect the presence of *Bartonella* species and to evaluate the genetic diversity of *B. henselae* strains by multiple locus VNTR analysis (MLVA). *Bartonella henselae* was the only species isolated from 36 (17%) of 211 cats. *B. henselae* genotype I was the predominant genotype (64%). MLVA typing of 259 strains from 30 bacteremic cats revealed 52 different profiles as compared to only 3 profiles using MLST. Of these 52 profiles, 48 (92.3%) were identified for the first time. One-third of the cats harbored one MLVA profile only. As there was a correlation between the age of cats and the number of MLVA profiles, we hypothesized that the single profile in these cats was the profile of the initial infecting strain. Two-third of the cats harbored 2 to 6 MLVA profiles simultaneously. The similarity of MLVA profiles obtained from the same cat, neighbor-joining clustering and structure-neighbor clustering indicate that such a diversity likely results from two different mechanisms occurring either independently or simultaneously: independent infections and genetic drift from a primary strain.

## Introduction

*Bartonella henselae* is a small, gram negative, arthropod-borne bacterium that has been shown to cause multiple clinical manifestations in humans, including cat scratch disease, bacillary angiomatosis, hepatic peliosis, hepatitis, endocarditis, fever and bacteremia [1], [2]. Three distinct genotypes have been defined on the basis of their 16S rDNA sequence [3], [4]. Genotype I has been associated with more severe clinical manifestations than those induced by genotype II in humans. The third genotype I/II strains (with both type I and type II 16S rDNA) is the less common [5].

Domestic cats are the major reservoir for this zoonotic agent. The transmission of *B. henselae* from cat to cat is ensured by the cat flea *Ctenocephalides felis* [6]. Ticks and biting flies have also been described as potential vectors [7], [8], [9].

Naturally infected cats develop persistent or recurrent bacteremia. Long lasting bacteremia ranges from 2 [10] to 5 months [11]. The fact that relapses can occur 18 months after the end of detectable bacteremia has led to suggest that infection could last more than 18 months without reinfection [12]. According to some studies, long lasting intra-vascular infections could be explained both by co-infections and by antigenic variations allowing *B. henselae* to escape the host immune response. Simultaneous detection of different *Bartonella* species and/or types of *B. henselae* have been described in the same individual [12], [13], [14], [15]. Antigenic variations have been identified during human infection by *B. quintana* [16]. At least one part of the antigenic variability could be related to the presence of tandem repeats within certain genes encoding some outer proteins [17], [18], [19].

Several molecular typing methods have been developed for *B. henselae*. Pulsed-field gel electrophoresis (PFGE), multilocus sequence typing (MLST) and multispacer typing (MST) techniques have been used for studying the epidemiology of *Bartonella* spp. and their genetic diversity [20], [21], [22], [23]. By combining PFGE and MLST, Berghoff *et al* [19] have shown that variants revealed by PFGE in primary isolates from one cat are closely related to each other and represent one strain.

Monteil *et al.* [24] have developed multiple locus variable number tandem repeats (VNTR) analysis (MLVA) for *B. henselae*. This technique is based on five polymorphic *B. henselae* variable number tandem repeats (VNTRs), named BHV-A to -E, for which numbers of basic units vary according to strains. Compared to the previously mentioned typing techniques, MLVA has proven to be the most discriminant method based on the value of the diversity index [25].

In this paper we report the results of a cross sectional study of *Bartonella henselae* bacteremia in stray cats from Algiers, Algeria. Strains were typed by MLVA in order to assess the genetic diversity of *B. henselae* among cats and compared to already described MLVA profiles [26]. When needed, some strains were also typed by MLST. The similarities between MLVA profiles were analyzed at the scale of each cat. Our results show that specific profiles are associated to the majority of these North African cats. Our results also suggest that strains diversity within cats may occur either by two or more independent infections (either concomitant or consecutive) or by genetic drift from a primary strain, sometimes in the same feline host.

## Materials and Methods

### Collection of Samples from Cats

All the cats introduced in this study were caught in the context of the National Program for Rabies Control in which the authors of the paper are not involved. This program is carried out by Hygiène Urbaine d'Alger (HURBAL), which is an institution trust of the Algerian Ministry of Interior, the Local Government and the Algerian Ministry of Agriculture and Rural Development. The Director of this establishment is a Veterinary Doctor. Two additional veterinary doctors are responsible for ensuring the good health of the animals that are caught, and take care of sick animals, giving them the required treatments. Their assistants have received training on animal welfare and on the careful methods to be used to capture animals. Cats are caught carefully with sheathed clamp. Once captured, the animals are housed in cages regularly cleaned and disinfected: maximum 10 cats (males or non pregnant females) per cage (size 2 m×2 m). Pregnant females are placed in individual cages (one cat per cage). Animals are inspected daily, fed (meat, milk) and given fresh water. They are euthanized only after expiration of the legal delay of guard (7 days, in order to allow owners to claim their animal). On 23 March 2008, one of us (NA), was given authorization by HURBAL to collect blood samples.

Between July 2008 and August 2010, blood samples were collected from 211 stray cats living in the city of Algiers, Algeria, and its suburbs. These stray cats were captured and, after sampling, humanely euthanized for stray cat population control. Sampling was conducted in the morning on anaesthetized cats (0.5 mg/Kg acepromazin (Vétranquil, Sanofi) and 15 mg/Kg ketamine (Immalgène 1000, Mérial)) in a room dedicated to and equipped for veterinarian activities. Euthanasia followed anaesthesia without time for awakening and was performed on anesthetized cats by intravenous injection of pentobarbital natrium overdoses. Under aseptic conditions, 5 ml of blood were collected by intracardiac puncture, and then transferred into separators (2 ml) and EDTA tubes (3 ml). Serum and whole blood were stored at −27°C until analysis.

The age of cats was estimated according to their physical aspect and to the erosion level of the permanent teeth. Five age classes were considered: <12 months, 12–17 months, 18–23 months, 24–35 months and ≥36 months. The pregnant status of females was determined by abdominal palpation.

### *Bartonella* Isolation

The EDTA treated tubes were centrifuged at 1800 g for 75 mn at room temperature. Three hundred µl of Schneider’s Drosophila medium (1x, L-glutamine, Gibco) were mixed with the pellet by vortexing. The suspension was first streaked onto 2 brain heart infusion agar media enriched with 5% fresh rabbit blood, then incubated at 35°C in a 5% CO_2_ atmosphere. The inoculated plates were examined daily for bacterial growth for 4 weeks [27].

The number of bacterial colonies was recorded for each bacteremic cat and colony-forming units (CFU) per milliliter of blood were calculated. Then, up to 14 colonies were randomly selected and sub cultured separately onto BHI agar supplemented with 5% fresh rabbit blood. For each bacteremic cat, 0.05% to 100% of isolated colonies were selected randomly. The subculture plates were incubated in the same culture conditions, as previously indicated, for 5–10 days.

### DNA Extraction of Single Colony Derived Culture

Colonies were scraped from the agar and suspended in 500 µl of sterile distilled water. The suspension was boiled for 10 min. After centrifugation (3000 g for 15 min), the cell lysate supernatants were stored at −25°C until tested by PCR.

### Identification of *Bartonella* Species and 16S rRNA Gene Typing

For the characterization of the isolates, PCR amplification of the 16S-23S intergenic sequences was performed as described by Jensen *et al.* [28]. The primer set used included B1623R (AACCAACTGAGCTACAAGCC) and JEN1F (CTCTTTCTTCAGATGATGATCC). The expected band size was 145 bp for *B. clarridgeiae* and 163 bp for *B. henselae* [29].

*B. henselae* 16S rRNA genotyping was performed according to Bergmans *et al.* [3]. The method is based on 3 bp differences between *B. henselae* genotypes I and II within the sequence of the 16S rRNA gene [3]. Two PCR mixes were prepared, using two sets of primers: 16SF and either BH1 or BH2. For some strains, the 16S rRNA typing results were confirmed by sequencing: amplification was performed using primer 16SF 5’ (AGA GTT TGA TCC TGG YTC AG) 3’ and a new designed reverse primer 16SR 3’ (GTC ACC CCT TAT AAC CTGC) 5’.

### MLVA for Comparison of *B. henselae* Isolates

In order to type our strains, five polymorphic VNTR loci (BHVA to BHVE) were tested using the method of Monteil *et al.* [24]. Briefly, each BHV PCR mix contained 5 µl cell lysate, 2.5 µl 10× Pfx amplification buffer, 1 µl of 10 mM DNTP mixture, 0.5 µl MgSO_4_(50 mM), 2 µl of each reverse and forward primers (10µM), 2.5 µl of 10X PCR enhancer solution and 0.4 µl platinum Pfx DNA polymerase (2.5 units/µl) (InVitrogen, California, USA). PCR amplification program consisted of a denaturation step at 94°C for 5 mn followed by 34 amplification cycles: 94°C for 30 s, 53°C for 30 s and 72°C for 2 mn, with a final extension step of 10 mn at 72°C. According to their sizes, the PCR products were separated in 1–2% agarose gels in 1X tris-borate buffer. Electrophoresis was performed in 30 cm gels using migration time lengths up to 27 hours in order to get better evaluation of the amplicon sizes.

The genetic diversity of *B. henselae* strains was evaluated by calculating the diversity index (DI), using Hunter and Gaston method [30]. The obtained MLVA profiles were compared to 114 profiles [24], [26] from other continents: Asia (n = 23), Australia-New Zealand (n = 6), Europe (n = 56) and North-America (n = 29). The links between profiles were analyzed using a Minimum Spanning Tree (MST). The priority rule for constructing MST was set in order that the type that had the highest number of single-locus variants (SLVs) would be linked first. A cut-off value of maximum differences of 1 VNTR out of 5 was applied to define clusters in the MST method.

### MLST Typing

In order to appreciate the distance between the genetic variants observed simultaneously using MLVA in some cats, and to compare the results obtained with MLVA *vs* MLST typing techniques, 12 stains isolated from 3 cats (# 71, 88 and 92) harboring the highest diversity of strains - as stated by both 16S rRNA and MLVA typing - were chosen. The eight genes were amplified by PCR according to Iredell *et al.* (21) and sequenced (Eurofins, Paris, France). Sequence types were defined as indicated on the Multi Locus Sequence Typing Web site (http://bhenselae.mlst.net/).

### Data Analysis

A bivariate analysis was performed to study the association between two co-variates (the age class and the gender/physiological status: male, pregnant female, non-pregnant female) and three variables of interest: the bacteremic status of cats, and, in bacteremic cats, the natural logarithm of the number of CFU/ml of blood as well as the number of MLVA alleles.

#### Similarity of MLVA profiles obtained from the same cat

The similarity of MLVA profiles obtained from the same cat was analyzed using a bootstrap method. Only cats for which >1 MVLA profile had been observed were taken into account. Null hypothesis was that two pairs of MVLA profiles obtained from the same cat should be as close as two pairs of MVLA profiles obtained from different cats. The distance between two MVLA profiles was the number of VNTR for which the number of repeats was different (distances thus varied between 0 and 5). The analysis was focused on the average of within-cat mean distances between MVLA profiles. The observed value of this statistic of interest was first computed. A resampling procedure was then used to simulate from the data, under the null hypothesis. The resampling procedure was based on the adjacency matrix that links cats (rows of the matrix) and MLVA profiles (columns of the matrix). A bootstrap sample was obtained by generating a random permutation of the matrix column headers (the MLVA profiles). This procedure guarantees that the marginal sums of the adjacency matrix are constant (i.e. number of profiles per cat, number of cats per profile), whereas the profiles are randomly redistributed. Ten thousand bootstrap samples were thus generated and, for each of these, the statistic of interest was calculated. The corresponding distribution was finally examined to determine the empirical p-value of the null hypothesis test: this p-value was the proportion of the samples (simulated under the null hypothesis) for which the statistic of interest was below the actual value (computed from the data). The whole analysis was simultaneously performed globally, and for each cat.

#### Cluster analysis

Two clustering methods were applied to the set of MLVA profiles: neighbor-joining and structure-neighbor. The distance used for neighbor-joining was the same as above (i.e. for two profiles, the number of VNTR for which the number of repeats was different). Tree confidence was estimated by standard bootstrapping with 100 pseudoreplicates. Structure-neighbor clustering was proposed by Hall and Salipante [32] as an alternative method to classical clustering methods (such as neighbour-joining) when the number of characters is low, as it is often the case in MLVA studies for species with few appropriate VNTR loci. Briefly, this method starts with a Bayesian step [33] to determine the most likely number of clusters, which produces for each individual, the posterior probability that it belongs to each cluster. Individuals are then affected to the most likely cluster, except if the corresponding posterior probability is <0.80, individuals being left unclassified in that case. Within each cluster, each individual is finally linked to its nearest neighbor(s), the link being labeled by the corresponding distance. The distance used throughout the whole procedure is the Manhattan distance (i.e. the mean number of repeat difference between two profiles).

Data analysis was performed using the R program [34] and with the software provided online by Hall and Salipante [32] for structure-neighbor clustering. Minimum spanning tree was calculated using the Bionumerics software package version 4.6 (Applied-Maths, Sont-Martens-Latem, Belgium).

## Results

### Description of the Cat Population

Of the 211 cats sampled, 84 (39.8%) were males and 127 (60.2%) were females. 31.5% (40/127) of females were pregnant. All cats were described as belonging to the European breed and age ranged from 8 to 36 months (mean 18 months, median 15 months) (Table 1).

**Table 1:** Distribution of *Bartonella henselae* in cats.

	Total No. ofcats (%)	No. of bacteremic cats (%)
**Gender**		
Male	84 (39.8)	17 (8.1)
Female :	127 (60.2)	19 (9.0)
Pregnant	40	4
Unpregnant	87	15
**Age (months)**		
<12	12 (5.7)	4 (1.9)
12–17	99 (46.9)	21 (10)
18–23	21 (10,0 )	4 (1.9)
24–35	64 (30.3)	7 (3.3)
> = 36	15 (7.1)	0
**Total**	211	36 (17)

### Isolation and Identification of *Bartonella* Species

*Bartonella* were isolated from 36 (17%) of the 211 cats. All were shown to be *B. henselae,* based on the size of their 16S–23S intergenic PCR fragment. This was confirmed by 16S rRNA genotyping and by MLVA (see *infra*). 14 cats (46.7%) were infected with *B. henselae* genotype I strain(s), 10 cats (33.3%) with *B. henselae* genotype II strain(s) and 6 cats (20%) harbored both genotypes. In this last group, only one strain presented the third genotype I/II, while the 5 other cats were co-infected by both genotypes. The levels of bacteremia ranged from 1 to 1500 CFU/ml of blood.

**Figure 1:**
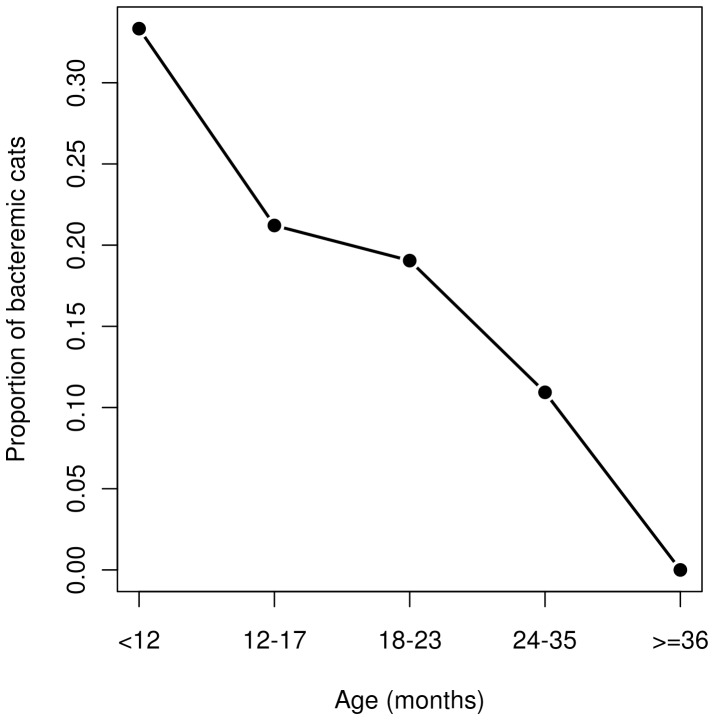
Association between the proportion of bacteremic cats and the age of cats.

The proportion of bacteremic cats showed a significant decreasing trend according to the age class (χ^2^ test for trends in proportions, p = 0.006), as one third of 12 cats less than one year old were bacteremic, whereas none of 15 cats ≥36 months old were bacteremic (Figure 1). No association was observed between the proportion of bacteremic cats and the gender/physiological status (Fisher’s exact test: p = 0.34).

No association was observed between the natural logarithm of the number of CFU/ml of blood and the age class (Kruskal-Wallis rank sum test: p = 0.91) or the gender/physiological status (Kruskal-Wallis rank sum test: p = 0.35).

### Diversity of the *Bartonella henselae* Strains from ALGIERS and Comparison of their Profiles to those of the Strains Already Typed by MLVA in Other Continents

**Figure 2:**
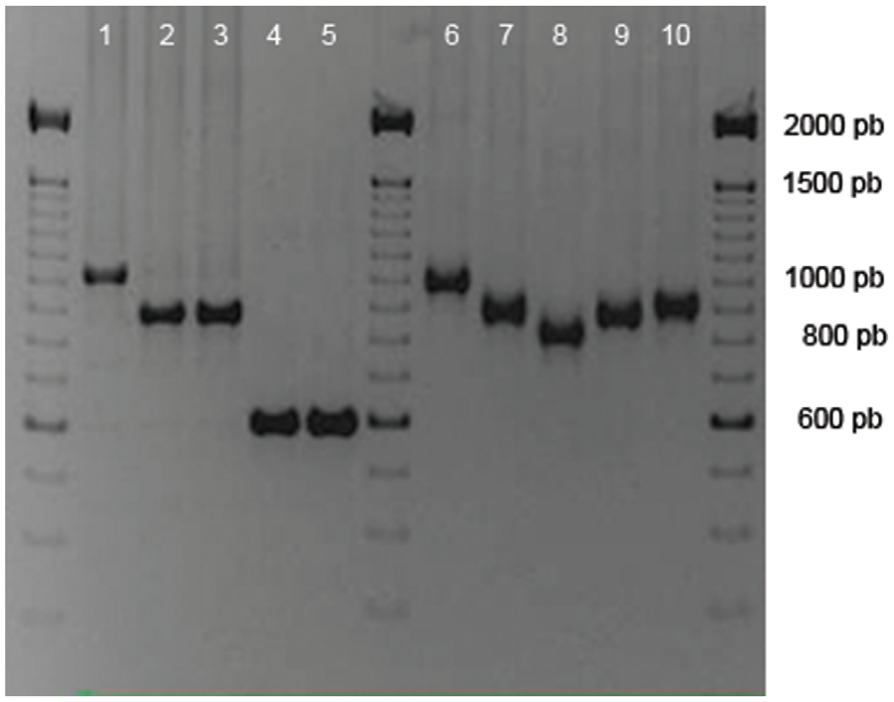
Polymorphism of BHV-A in 10 *B. henselae* strains by agarose gel electrophoresis. Lanes number 1 to 10: *B. henselae* strains isolated from cat # 92.

A collection of 259 strains of *B. henselae* was obtained from 30 bacteremic cats. The isolates of 6 remaining cats were lost during sub culture. All were analyzed by MLVA (Figure 2) leading to 52 different MLVA profiles (Table 2 and Table 3).

**Table 2:** Description of *B. henselae* profiles.

Allelic profile(BHV-A.-B.-C.-D.-E)	N° of MLVA profiles	16s rRNA genotype	Cat No
7.10.10.8.2	1	I	88
7.20.8.8.2	1	I	81
7.20.10.8.2	2	I	88
7.34.10.7.3	1	I	138
8.20.8.8.2	6	I	81
8.20.10.8.2	21	I	134, 92, 204
8.34.10.7.3	4	I	138
9.17.6.7.3	1	II	267
9.18.5.7.4	15	II	51, 88, 247
9.18.6.7.3	4	II	267
9.18.6.7.4	9	II	51, 142, 247
9.18.10.8.2	1	I	273
9.20.5.8.2	4	I	91
9.20.6.7.4	1	II	9
9.20.6.8.4	1	II	9
9.20.8.8.2	3	I	81
9.20.10.8.2	41	I, I/II	50, 79, 92, 127, 37, 242
9.25.5.8.4	1	II	237
9.31.10.7.3 *	6	I	237
9.32.10.7.3	11	I	232
9.34.10.7.3	3	I	138
10.18.5.7.4	1	II	88
10.18.6.7.3	2	II	267
10.20.6.7.4	6	II	9, 88
10.20.10.8.2	17	I	37, 71, 204, 79, 249,92
10.24.6.8.4	1	II	92
10.25.10.8.4	3	II	92
10.34.10.7.3	1	I	138
11.18.5.7.4	7	II	33
11.18.6.7.4	1	II	33
11.18.11.9.2	1	I	33
11.20.5.8.2	4	I	91
11.20.6.7.4	3	II	9
11.20.6.8.2	2	I	91
11.20.7.8.2	1	I	91
11.20.10.7.4	1	II	273
11.20.10.8.2	11	I	71, 134, 204
11.20.10.8.4	10	I	104
11.24.6.8.2	1	II	92
11.24.6.8.4	1	I+II	92
11.25.10.8.4	8	II	28
11.32.10.7.3	1	I	232
11.34.10.7.3	1	I	138
12.18.6.7.4	1	II	247
12.28.8.7.4	9	II	55
12.32.8.7.4	1	II	40
13.32.8.7.4*	3	II	40
14.20.10.8.2*	11	I	43, 127
14.26.8.7.4	4	II	71
14.27.10.7.4	1	II	67
14.32.8.7.4*	6	II	40, 191
15.32.8.7.4	2	II	40


* : profiles shared by Algerian strains and strains from other part of the World.

Allelic profiles are defined by the number of repeats at the five VNTR loci.

The overall Hunter and Gaston diversity index was 0.95, with a value of 0.90 for genotype I strains and of 0.94 for genotype II strains.

**Table 3:** Distribution of MLVA genotypes within the *B. henselae* bacteremic cats.

Cat No	No of colonies tested by cat	16S rRNA Genotype		No. of MLVA type by cat
		I	II	
9	10	0	10	4
28	8	0	8	1
33	9	1	8	3
37	11	11	0	2
40	9	0	9	4
43	10	10	0	1
50	10	10	0	1
51	5	0	5	2
55	9	0	9	1
67	1	0	1	1
71	14	10	4	3
79	10	10	0	2
81	10	10	0	3
88	8	4	4	5
91	11	11	0	4
92	10^(1)^	3	6	6
104	10	10	0	1
127	2	2	0	2
134	10	10	0	2
138	10	10	0	5
142	5	0	5	1
191	3	0	3	1
204	14	14	0	3
232	12	12	0	2
237	7	6	1	2
242	12	12	0	1
247	12	0	12	3
249	8	8	0	1
267	7	0	7	3
273	2	1	1	2
Total:30	259	166	93	72^(2)^

B.U.: Basic Unit.

(1) Including one strain with both 16S rRNA gene/genotypes.

(2) Among which 52 MLVA profiles were different (including the unique profile of the strain harbouring both 16S rRNA gene/genotypes).

Twenty five of these 52 MVLA profiles were displayed by strains belonging to genotype I (49%), 26 to genotype II (ratio genotype I/II  = 25/26) and one corresponded to the genotype I/II strain. There was no common profile to the three genotypes.

**Figure 3:**
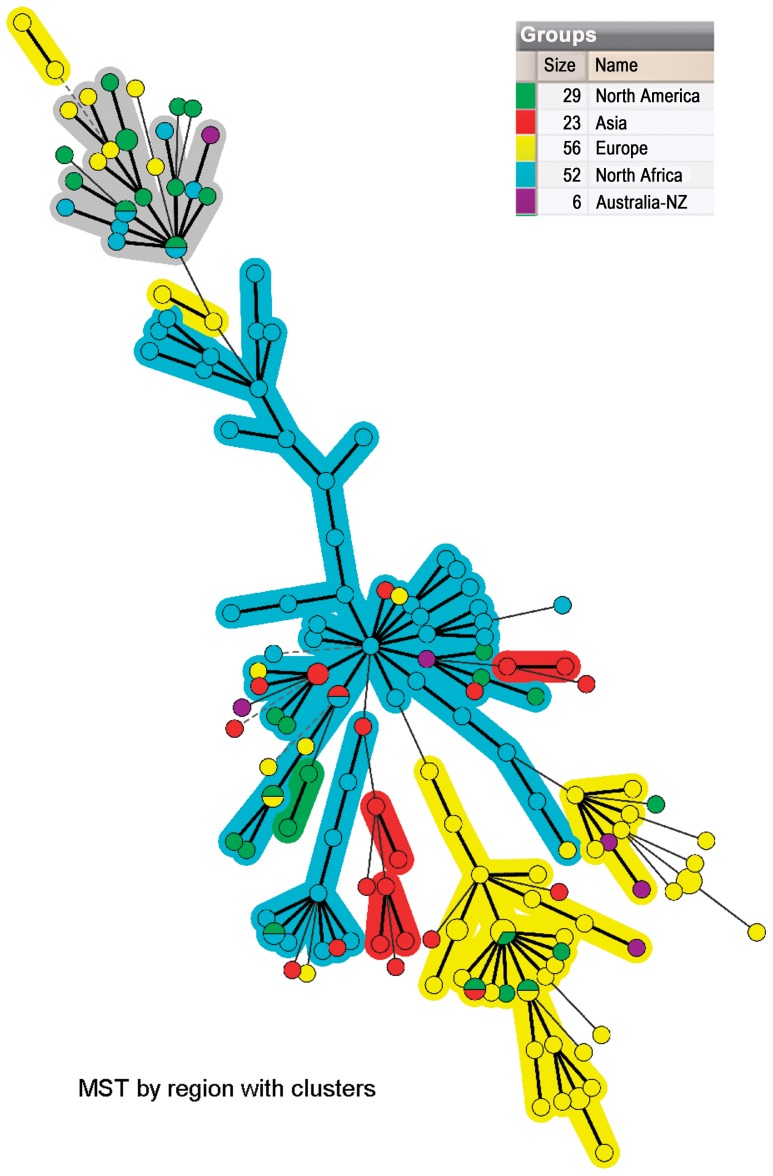
Minimum Spanning Tree of 52 *B. henselae* strains from Algiers and 114 strains from other part of the World on categorical analysis of 5 VNTRs. Each circle represents a unique genotype. The diameter of each circle corresponds to the number of strains with the same genotype. Genotypes connected by a shaded background differ by a maximum of 1 of the 5 VNTR markers. Thick connecting lines represent one marker difference; regular connecting lines represent two marker differences; thick interrupted lines represent three differences. The length of each branch is also proportional to the number of differences. Each geographical situation is represented by a specific colour: red for strains collected in Asia, yellow for strains collected in Europe, green for strains collected in North-America, purple for strains collected in Australia and blue for strains collected in North-Africa.

These percentages and ratios for the 51 profiles of Algerian strains corresponding to one clearly identified 16S genotype (49%, 25/26) were intermediate between those of the Asian (87%, 20/3) and European (14%, 8/48) strains (Table 4). The minimum spanning tree (MST) (Figure 3) confirmed that the Algerian strains form a rather homogenous group at the center of the tree, among the rest of the strains (from Europe, Asia and the USA). The proportions of genotype I *vs* II genotypes were significantly different according to the world region of origin (Fisher's exact test, p<0.0001).

**Table 4:** Comparison of distribution of *B. henselae* genotypes of Algerian strains or profiles and strains from other countries/region of the world (this study and Bouchouicha *et al.* [26], Monteil *et al.* [24]).

	Number of MLVA profiles	Genotype I	Genotype II	Proportion I/II	BHV A mean of repeats	BHV A sd	BHV A min	BHV A max
Algeria	51	25	26	0.49	10.24	1.91	7	15
Asia	23	20	3	0.87	13.13	1.98	9	16
Europe	56	8	48	0.14	11.52	2.11	9	15
	**BHV B mean of repeats**	**BHV B sd**	**BHV B min**	**BHV B max**	**BHV C mean of repeats**	**BHV C sd**	**BHV C min**	**BHV C max**
Algeria	23.22	6.07	10	34	8.00	2.00	5	11
Asia	23.17	9.48	6	37	10.48	5.79	1	20
Europe	16.11	6.57	6	33	5.00	3.76	2	18
	**BHV D mean of repeats**	**BHV D sd**	**BHV D min**	**BHV D max**	**BHV E mean of repeats**	**BHV E sd**	**BHV E min**	**BHV E max**
Algeria	7.47	0.54	7	9	3.12	0.89	2	4
Asia	5.65	3.27	1	9	2.52	0.67	1	3
Europe	3.84	2.88	1	12	2.61	1.52	1	6

Most of the MVLA profiles were new (48/52, 92.3%), as only 4 profiles were common with profiles of strains isolated in other countries.

In the Algerian cats, the number of MLVA profiles obtained from a given cat increased with age and was associated with the age group of the cats (Kruskal-Wallis rank sum test: p = 0.02), increasing from a mean number of profiles of 1 in cats less than 1 year old to a mean of 3.2 MLVA profiles in cats 24 to 35 months old. No association between the number of MLVA profiles and the gender or physiological status of the cats was observed (Kruskal-Wallis rank sum test: p = 0.93).

BHV-A (9 alleles) and BHV-B (12 alleles) were the most polymorphic VNTRs, followed by BHV-C (6 alleles), BHV-D (3 alleles) and BHV-E (3 alleles) (Table 2). Data concerning the alleles and number of repetitions (mean, standard deviation, Min, Max) for each locus/BHV are presented in Table 4. The range of the number of repetitions for BHV A (intergenic) was similar in Algerian strains to that of Asian and European strains. Conversely, the ranges and standard deviations for the 4 intragenic VNTR, BHV B, C, D and E in Algerian strains were narrower than those observed in Asian or European strains.

In the case of BHV-C and E, all the alleles (2, 6 & 10 for BHV-C and 2, 3 & 4 for BHV-E) but one (allele 1 for BHV-E) previously identified in *B. henselae* strains from other regions of the world (e.g. alleles 2 & 6 for BHV-C in Europe *vs* allele 10 in Asia; allele 4 for BHV-E in Europe *vs* alleles 2 & 3 in Asia) were also present in our population of Algerian strains (Table 5).

**Table 5:** Comparison of alleles of BHV-C and BHV-E associated to world region origin (this study and Bouchouicha *et al.* [26], Monteil *et al.* [24]).

Dominantallele(s) for	Europe	North America	Asia	Australia/NZ	Algeria	
		East	West			
BHV-C	2 or 6	2 or 6	10 or 2	10	10	10 or 6
	(74–83%)			(60–100%)		
	10	(58%)	(64%)	2 or 6	(83%)	(72%)
	(0–8%)			(0–15%)		
BHV-E	1 or 4	1 or 4	1 or 4	3 or 2	10	2 or 4 or 3
	(71–100%)			(80–100%)		
	3 or 2	(58%)	(77%)	1 or 4	(83%)	(100%)
	(0–12%)			(0–20%)		

(%): percentage of strains with a given allele.

### Similarities between MLVA Profiles Obtained from the Same Cat

**Figure 4:**
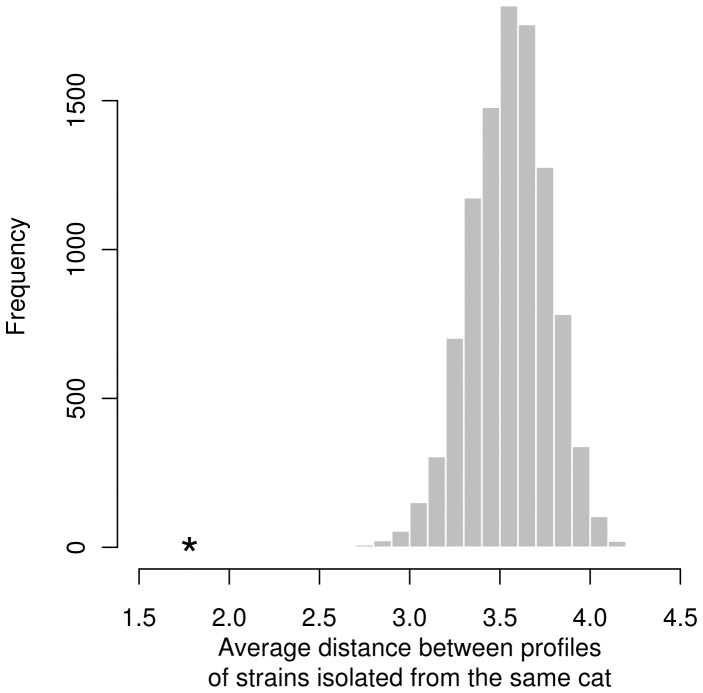
Distribution average distance between profiles of strains isolated from the same cat. Observed average distance between MLVA profiles obtained from the same cat (star) and distribution of simulated values under the null hypothesis that two pairs of MVLA profiles obtained from the same cat are as similar as two pairs of MVLA profiles obtained from different cats.

Among the 30 cats tested by MLVA, 20 displayed more than one MLVA profile (Table 3). The similarities between the different MLVA profiles obtained from a given cat were analyzed for those 20 cats harboring 2 to 6 different MLVA profiles. In these 20 animals, the MVLA profiles observed in a given cat differed by 1.8 VNTRs on average. According to the animals, the value of this statistic varied between 1 and 4 (Table 6). In the 10,000 bootstrap samples generated under the null hypothesis, the average of cat-specific mean distances between MVLA profiles was always above the observed value of 1.8 (Figure 4). Therefore, the null hypothesis could be rejected with a high level of confidence (p<0.0001): two pairs of MVLA profiles obtained from the same cat appeared significantly more similar than two pairs of profiles obtained from different cats. However, for four (cats # 71, 88, 237 and 273) of the 20 cats, the cat-specific p-value was >0.05 (Table 6).

**Table 6:** Observed average distance between MLVA profiles obtained from each cat.

Cat number	Average distance^1^ between MLVA profiles	p^2^
127	1.0	<0.0001
134	1.0	<0.0001
138	1.0	<0.0001
142	1.0	<0.0001
204	1.0	<0.0001
232	1.0	<0.0001
237	4.0	0.42
247	1.3	0.001
267	1.3	0.001
273	4.0	0.43
33	2.3	0.04
37	1.0	<0.0001
40	1.0	<0.0001
51	1.0	<0.0001
71	3.7	0.41
79	1.0	<0.0001
81	1.0	<0.0001
88	3.6	0.45
9	1.3	<0.0001
91	1.3	0.0001
92	2.7	0.004

1 Mean number of loci for which the repeat number is different, averaged over all profile pairs.

2 Approximate p-value determined using a bootstrap method.

Approximate p-value is obtained testing the null hypothesis that two pairs of MVLA profiles obtained from the same cat are as similar as two pairs of MVLA profiles obtained from different cats.

### MLST Analysis

As shown in table 7, only two ST per cat were observed in the case of the three cats that were tested.

**Table 7:** MLST typing of 12 strains.

Allelic profile (BHV-A.-B.-C.-D.-E)	Sequence Type
Cat#71	
11.20.10.8.2	ST1
14.26.8.7.4	ST5
10.20.10.8.2	ST1
Cat #88	
9.18.5.7.4	ST5
10.18.5.7.4	ST5
7.20.10.8.2	ST1
Cat#92	
11.24.6.8.2	ST24
8.20.10.8.2	ST1
10.25.10.8.4	ST24
10.24.6.8.4	ST24
9.20.10.8.2	ST1
9.24.6.8.4	ST24

### Cluster Analysis

#### Neighbor-joining clustering

**Figure 5:**
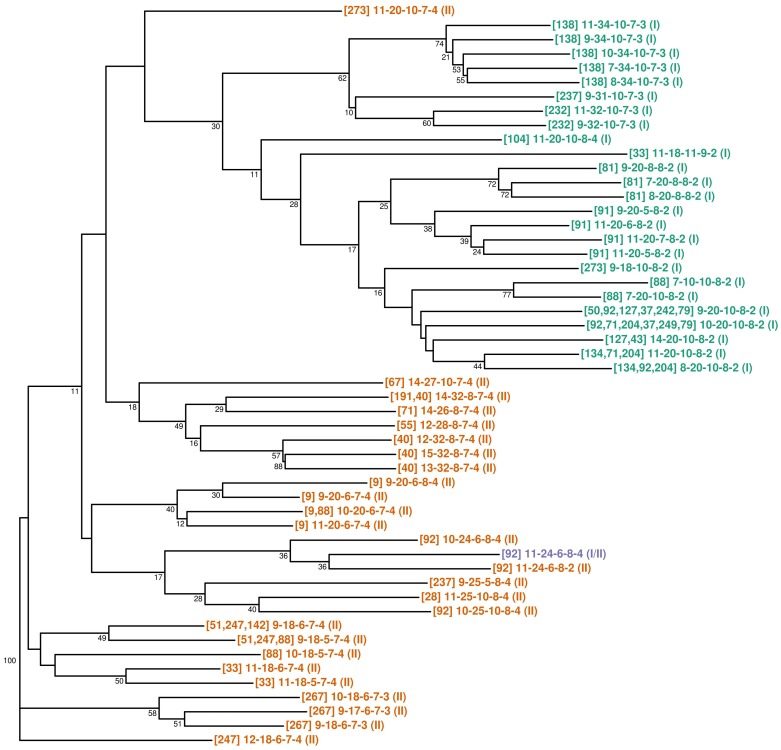
Dendrogram of the profiles generated by Neighbor Joining. Each profile is identified by: [Number of Cat(s)] VNTR profile (16SrRNA genotype). Frames identify *B. henselae* 16S rRNA type II.

A total of 259 strains from 30 cats and 52 VNTR profiles were considered for this analysis. The dendrogram (Figure 5) clearly separates the MLVA profiles from strains belonging to genotype I and genotype II. As previously reported [32], few clades had a >70% bootstrap confidence. Due to the low number of characters (n = 5 loci), MLVA data did not contain sufficient information to infer reliable relationships among MLVA profiles. Nevertheless, cat-specific profiles appeared grouped for some of the cats such as animals # 40, 81, 91, 138 and 267 (Figure 5). In these cats, the analysis of the profiles similarity revealed a significant similarity supported by low p-values (Table 6, p<0.001) Conversely, for 4 cats (# 71, 88, 237 and 273), the different strains isolated from each cat, which also belonged to different 16S rRNA genotypes, were not grouped in the tree. In these cats, the analysis of the profiles similarity did not revealed any similarity, characterized by high p-values (Table 6, p>0.4).

#### Structure Neighbor clustering

**Figure 6:**
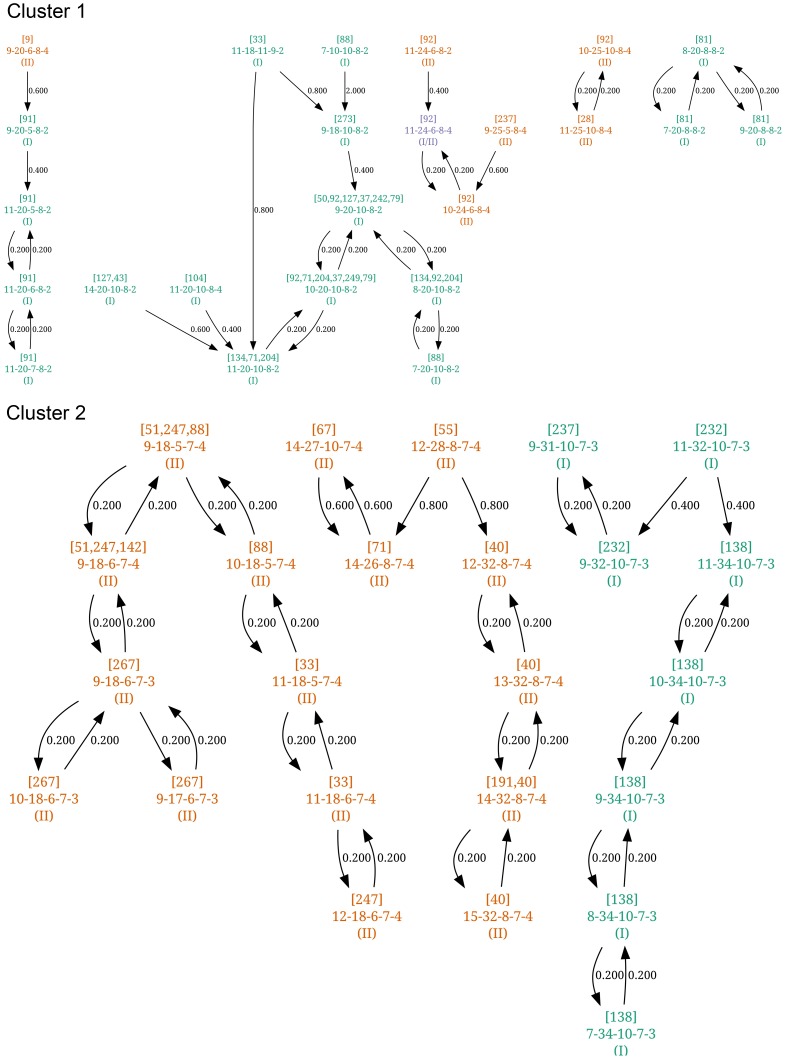
Structure-neighbor clustering analysis of MLVA profiles. Two clusters are defined. Within each cluster, each profile is connected to its nearest neighbor(s) and the corresponding distance is indicated (the mean difference between the number of repeats). Each profile is identified by: [Number of Cat(s)]/VNTR profile/(16SrRNA genotype).

Two clusters were defined by structure-neighbor clustering. Twenty-four profiles were assigned to cluster 1 and 24 to cluster 2, and 4 profiles could not be classified. The diagram of nearest neighbors for profiles in the two clusters is presented in Figure 6, indicating that 16S genotypes I and II were present in both clusters. However, the different profiles of strain isolated from the same cat always belonged to the same cluster except for 4 cats (# 71, 88, 237 and 273) infected by strains belonging to both clusters. Furthermore, cat-specific profiles appeared grouped for some individuals, such as cats # 40, 81, 91, 138 and 267 (Figure 6). These results were also in agreement with those obtained by neighbor-joining clustering (Figure 5), and with results from the analysis of the cat-specific profiles similarity (Table 6).

## Discussion

This cross sectional study of *Bartonella* infection in stray cats from Algiers allowed us to study the distribution and diversity of *Bartonella henselae* strains among these North African cats using MLVA. Our data support that *B. henselae* strain diversity in a given cat results from independent infections (either concomitant or consecutive) co-infection and/or genetic drift.

This is the first report of the isolation of *Bartonella henselae* in cats from North Africa. In our study, 17% (36/211) of cats were bacteremic. All the 259 isolated strains were identified as *B. henselae*. The prevalence of bacteremic stray cats was found to be in the range of previous studies in other parts of the world [20], [35], [36], [37], [38], [39]. Despite the approximate evaluation of the age of the cats, we were able to show that the proportion of bacteremic cats was higher in younger animals, as previously reported [11], [40], [41]; but no correlation was established between the bacteremia level and the age of the bacteremic cats. Similarly, no correlation was demonstrated between the proportion of bacteremic cats and pregnancy. All 259 strains belonged to *B. henselae* and no *B. clarridgeiae* was isolated. This result confirms the difficulty to isolate *B. clarridgeiae* and suggests that *B. clarridgeiae* may be better adapted to its vector than to mammalian hosts [9].

The 259 strains analyzed by MLVA were distributed in 52 profiles, of which 48 were new profiles, when compared to those obtained in our previous study [26]. Taken together, these two studies led to identify 147 original profiles for 437 strains.

As in our previous study, the high number of original profiles is to be linked to the fact that the population of cats tested belonged to a new region of the world being investigated. The HG diversity index calculated for the 259 strains was a little bit lower than that obtained in our precedent study (0.95 *vs* 0.98), which may be explained by the selection of the strains, one per cat in the previous study to up to 14 strains per cat in this study. Nevertheless, a D.I. of 0.95 is considered as excellent [42].

The use of MLVA at the scale of individual cats allowed distinguishing two groups. The first group was constituted of 20 cats that were infected by more than one variant/profile. The NJ dendrogram showed that all the profiles belonging to 16SrRNA type I were grouped within one clade. The genotype II profiles had a more scattered distribution. Analysis of the cat-specific profiles similarities indicated that the strains/profiles isolated from one cat are more similar to each other(s) than those taken randomly from the Algiers cat population (Figure 4). This result was confirmed by structure neighbor clustering. This approach led to dispatch the profiles of the strains/variants isolated from a given cat in one of the two defined clusters. However, four cats (# 71, 88, 237, and 273) did not fit with one or more of these analyses. They belonged to the series of 6 cats that were co-infected by the two genotypes. The three statistical analyses used in the present study strongly suggest that different profiles isolated from a given cat originated from either independent infections by two or more different strains or by genetic variation (drift) from the same initial stain. In a few instances, both events seemed to occur simultaneously in a specific cat. As there is a correlation between the number of VNTR profiles and age, we could say that co-infection occurred in a specific cat, e.g. for strains belonging to different 16S genotype as well as for strains belonging to the same genotype but with distant MLVA profiles (for example, strains isolated from cat # 92, Figure 6). This hypothesis was partially confirmed by the fact that the number of bacteremic cats decreased with age whereas the number of profiles they harbor increased. Our results using MLVA only confirmed the results obtained previously on a smaller number of strains by Berghoff *et al.* [19], with both PFGE and MLST. More diversity of MLST profiles was observed by Berghoff *et al.* [19]. However, our study and that of Berghoff et al [19] are not completely comparable. First they used multiple single-colony-derived cultures rather than multiple primary isolates from the same blood sample in our case. Second, the geographical origins of their cats are much more diverse. As expected MLST differentiated 16S rRNA genotype I *vs* II strains within the same cat. But it was poorly discriminant at the scale of the strains tested when they belonged to the same 16SrRNA genotype. All genotype I strains belonged to the very common ST1 even when they were differentiated by MLVA. This is coherent with the greatest stability of MLST and with our hypothesis that from one cat to another (71 and 88) and even within one cat (92), new strains preferentially emerge by changing the number of basic units within the less stable VNTR, BHV-A, which is the only one to be intergenic.

In the case of 16S rRNA genotype II strains, MLST profiles were also found to be identical within a given cat. Two cats harboured the same common ST5, whereas cat 92 presented for all genotype II strains (with 4 MLVA profiles) a very rare MLST profile, ST24. As for cat 92, very different MLVA profiles were observed, our result suggests that either MLST is not predictive of the way the cats acquire strain diversity (drift vs infection by different strains) or that potentially the different polymorphic VNTR could be prone to strong variability, at a very short time scale, in circumstances that remain to be determined.

Eventually, the clearly more discriminatory power of MLVA as compared to MLST in our study, in addition to the data presented by Bouchouicha et al [25], strongly suggest that MLVA could advantageously replace MLST for epidemiological purposes whereas MLST remains the gold standard for phylogeny.

The second group included 10 cats infected by a single profile, with 3 to 12 colonies analyzed per cat. The proportion of cats infected by a single variant/profile only was lower than that obtained by Berghoff *et al.* [19] which is surprising, as PFGE is known to be a more sensitive technique for studying genetic variants in other bacteria. However, the two techniques converged to confirm that the cats were infected by a single variant/profile. As there was a correlation between the number of VNTR profiles and age of the cats, we hypothesized that the unique profile/variant in such cats was the profile of the initial infecting strain inoculated by flea(s). Such an infection by a single variant could result either from its emergence from a *B. henselae* cluster within a given cat colony or cat litter or from selective pressure within its flea vector, - a flea selecting the strain/variant to be transmitted among those ingested with a blood meal - or as passive filters – the flea being a site of strains/variants competition. This latter hypothesis is in agreement with the one formulated at the species level by Tsai *et al.* [9], who suggested that there is a discrepancy between the ability for a flea to harbor several species of *Bartonella* and this flea’s ability to transmit all these *Bartonella* species.

The diversity of profiles detected in cats infected with one variant/profile appeared similar to the diversity of profiles isolated in cats infected with different MLVA profiles, suggesting that all profiles were at equal chance to vary. Eight MLVA profiles isolated from cats infected with one variant/profile were also shared by cats infected by different variants/profiles. The absence of correlation between bacteremia levels and the number of VNTR profiles leads to the hypothesis that the presence in a same cat of one or several MLVA profiles does not depend on the *B. henselae* strain but on biological factors inherent to the mammalian host. Therefore, we tested half (15/30) of the cats for FIV and FeLV infections (data not shown). These viruses are known to be immunosuppressive and Buchmann *et al* [31] demonstrated that FeLV infection modifies the course of *B. henselae* infection of cats. In our cohort, only one cat was positive for FIV and none for FeLV infection. We can conclude that the presence of more than one variant in one cat is not the consequence of the immunodeficiency induced by these viruses.

This study underlines also some particularities of Algerian *B. henselae* strain diversity when compared to the other continents (Asia, Europe, USA). Half of the bacteremic cats were infected with *B. henselae* 16S rRNA genotype I. The proportion of genotype I strains in Algerian cats was intermediate between that observed in Asian feline populations and European feline populations [1]. When the two VNTR (BHV-C and BHV-E), previously recognized as geographic markers [26], were considered, it appeared that almost all the alleles that are dominant in Asian or European *B. henselae* strains world were also dominant in the Algerian strains. The allelic diversity of 4 VNTR: BHV-B, C, D and E, estimated by the range of the number of repeats of Algerian strains was lower than for Asian and European strains. Conversely, the range of the number of repeats in BHV-A, the only one among the five VNTR tested to be intergenic, was similar to that observed in the European or Asian *B. henselae* strains. Interestingly, the positioning of the Algerian strains in the MST suggests that they are located at the root of the strains originating from the other parts of the world, which were tested previously by our team.

As repeated sequences are involved in genetic diversity, especially intragenic repeats, and in the genesis of antigenic variability [17], [19], [43], it could be suggested that *B. henselae* has co-evolved with its feline reservoir host, and more generally with its ecosystem, from the moment domestic cats emerged in the Middle East [44]. Further studies, especially on Egyptian feline mummies, using MLVA typing, may allow investigating an ancestral diffusion to North Africa of both feline breeds and their *Bartonella* strains, characterized by more stable MLVA than among felines dispersed on other continents. Such a study would contribute to the understanding of co-evolution of cats, cat fleas and *B. henselae.*

### Conclusions

This survey on the prevalence of *B. henselae* infection in Algerian stray cats adds one more region in the world to those where cats have been investigated as reservoirs for the zoonotic bacterium *B. henselae*. The genetic diversity of isolated strains can result from co- infection and/or genetic drift. The occurrence of such mechanisms and their possible involvement in long lasting bacteremia will have to be taken into account for future vaccine design.

Our results suggest that cats are initially infected either by only one strain with further genesis of variants or can be co-infected by distinct strains from various cat fleas or potentially harbored by the same flea. The analysis of the MLVA results and the comparison of the alleles of 5 selected VNTR with *B. henselae* strains obtained from other parts of the world, lead to propose a co-evolution of the Cat/Flea/*B. henselae* system starting from the origin of the domestic cat, in the Middle East.
